# Acute Respiratory Infections in Travelers Returning from MERS-CoV–Affected Areas

**DOI:** 10.3201/eid2109.150472

**Published:** 2015-09

**Authors:** Matthew German, Romy Olsha, Erik Kristjanson, Alex Marchand-Austin, Adriana Peci, Anne-Luise Winter, Jonathan B. Gubbay

**Affiliations:** Public Health Ontario, Toronto, Ontario, Canada

**Keywords:** Respiratory tract infections, air travel, MERS, MERS-CoV, Middle East respiratory syndrome, travel, influenza, coronavirus, viruses, Canada

## Abstract

We examined which respiratory pathogens were identified during screening for Middle East respiratory syndrome coronavirus in 177 symptomatic travelers returning to Ontario, Canada, from regions affected by the virus. Influenza A and B viruses (23.1%) and rhinovirus (19.8%) were the most common pathogens identified among these travelers.

Middle East respiratory syndrome coronavirus (MERS-CoV) was originally described in 2012 in a patient with severe pneumonia in Saudi Arabia (*1*). The virus has been detected in several countries of the Middle East, causing acute respiratory disease and having a case-fatality rate of ≈35% (*2*). Although the exact epidemiology and mode of transmission remains ill-defined, MERS-CoV appears to be transmitted through respiratory droplets and most likely has zoonotic reservoirs in dromedary camels and possible origin in bats (*1*). Recent evidence suggests human infection results from repeated introduction of the virus from camels to humans, and less severe human-to-human transmission probably requires close contact with infected persons (*2*,*3*).

As of June 16, 2015, the World Health Organization (WHO) reported 1,293 laboratory-confirmed cases of MERS-CoV, of which 458 (35.4%) were fatal, with ongoing transmission in Saudi Arabia, an outbreak in South Korea and an imported case in Thailand (*2*). Reported cases are centralized in and around the Arabian Peninsula (Saudi Arabia, United Arab Emirates [UAE], Iran, Jordan, Kuwait, Lebanon, Oman, Qatar, and Yemen); Saudi Arabia and UAE account for ≈95.8% of cases (*2*). Internationally, imported cases have been reported outside this zone (United Kingdom, France, Germany, Tunisia, Italy, Malaysia, Philippines, Greece, Egypt, United States, the Netherlands, Algeria, Austria, and Turkey) (*2*). Within Saudi Arabia and UAE, cases are predominantly localized to Jeddah, Riyadh, and Abu Dhabi, each of which operates a high-traffic airport that serves 17–26 million international travelers each year (*4*,*5*). To detect imported MERS-CoV cases, public health authorities in Ontario, Canada, advise testing of persons who have acute respiratory infection (ARI; i.e., symptoms and signs consistent with acute upper or lower respiratory tract infections) of any severity and recent travel to MERS-CoV–affected areas or of persons with ARI and recent close contact with ill travelers from affected areas (*6*).

Peak travel periods to Saudi Arabia (e.g., Ramadan, Umrah, or the Hajj) are of particular concern, although after the 2012 and 2013 Hajj, no MERS-CoV cases were identified in persons returning to France (*7*). High incidences of other respiratory diseases in pilgrims varied by year. In this study, we aimed to explore the array of respiratory pathogens in travelers with ARI returning to Ontario from MERS-CoV–affected areas or in their close symptomatic contacts.

## The Study

During November 2012–June 2014, a total of 177 international travelers returning to Ontario were considered persons under investigation (PUIs) for MERS-CoV, according to the guidelines of the Ontario Ministry of Health and Long-Term Care (*6*). PUIs were recommended to be isolated and screened for MERS-CoV and other respiratory pathogens (*6*).

Nasopharyngeal swab samples and, for persons on ventilators, bronchoalveolar lavage specimens were collected from patients and submitted to Public Health Ontario Laboratories (PHOL), the provincial reference laboratory for MERS-CoV testing (*6*). Fecal specimens were collected when patients had diarrhea, and urine was collected during early phases of the outbreak when appropriate specimen collection standards were ill-defined (*6*).

MERS-CoV real-time reverse transcription PCR (rRT-PCR) targeted regions upstream of the E gene and within open reading frame 1b, as recommended by WHO (*8*). Influenza rRT-PCRs targeted the influenza A matrix gene and influenza B nonstructural 1 gene using Centers for Disease Control and Prevention (CDC; Atlanta, GA, USA) protocols. If the rRT-PCR was positive for influenza A virus, we conducted subtyping for seasonal influenza A(H3N2) virus hemagglutinin gene (CDC assay) and influenza A(H1N1)pdm09 virus neuraminidase gene (in-house assay) (*9*). Respiratory specimens were further tested by using Seeplex RV15 ACE multiplex respiratory viral assay (Seegene Inc., Seoul, South Korea). Targets included human rhinovirus, enterovirus, influenza A and B viruses, parainfluenza viruses 1–4, respiratory syncytial virus A and B, adenovirus, bocavirus, human metapneumovirus, human coronavirus OC43, and human coronavirus 229E/NL63. *Mycoplasma pneumoniae* and *Chlamydophila pneumophila* testing was conducted by using ProPneumo-1 multiplex assay (Gen-Probe Inc., San Diego, CA, USA). PCR was conducted for *Legionella* species by using a protocol developed by CDC (*10*); BinaxNOW Legionella Urinary Antigen Test (Binax Inc., Portland, ME, USA) was also conducted to test for *L. pneumophila* serogroup 1.

Of 177 PUIs (mean age 48.1 years, range <1–88 years; 56% male), 54.8% returned from Saudi Arabia or UAE. Identification of PUIs peaked after the October 2013 Hajj and after the first 2 MERS-CoV cases were imported into the United States in May 2014 (Figure). All PUIs had ARI; of the 85 PUIs for whom data were available, 47 (55%) and 74 (87%) had respiratory specimens collected within 5 and 14 days (median 4 days) from symptom onset, respectively. Specimens collected were as follows: 185 upper respiratory, 10 lower respiratory, 98 urine, 97 blood, 11 fecal, and 1 pleural fluid. Symptom onset varied from 17 days before return to 10 days after return (median <1 day after return) for the 20 PUIs for whom this information was supplied. One patient was excluded from the time-to-collection analysis because the specimen was collected under extenuating circumstances: testing was conducted because of worsening respiratory symptoms beginning 57 days before the patient returned from overseas.

**Figure:**
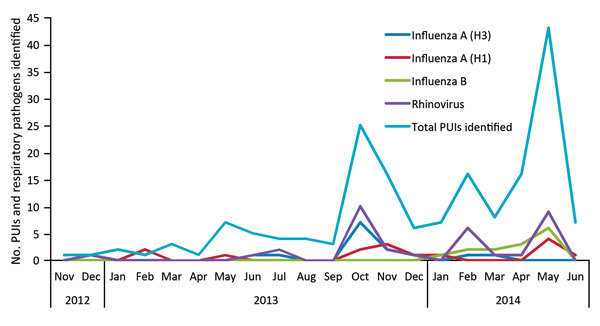
PUIs and counts of major respiratory pathogens identified in travelers returning to Ontario, Canada, from countries affected with Middle East respiratory virus coronavirus, December 2012–June 2014. PUI, persons under investigation.

At least 1 respiratory pathogen (bacterial or viral) was detected in 89 (50.3%) PUIs; however, for most (87 [98%] of 89) patients, only viral pathogens were identified (Table). Influenza was the most common virus identified: 27 (15.3%) persons tested positive for influenza A, 14 (7.9%) for A(H3N2) and 13 (7.3%) for A(H1N1)pdm09; 14 (7.9%) tested positive for influenza B. Rhinovirus was also common, detected in 35 (19.8%) persons, with a peak in the fall, in keeping with its seasonality in Canada (Figure; Table). Similarly, influenza A(H3N2) peaked in the fall, whereas influenza B and A(H1N1)pdm09 peaked in late spring.

**Table:** Respiratory pathogens detected among 177 persons tested for MERS Co-V at Public Health Ontario Laboratories, Ontario, Canada, November 2012–June 2014*

Pathogen†	Case count		
	No. (%)‡	Highest no. imported in 1 mo	Time of highest no.
Influenza viruses			
Influenza A (H3) virus	14 (7.9)	7	2013 Oct
Influenza A(H1N1)pdm09 virus	13 (7.3)	4	2014 May
Influenza B virus	14 (7.9)	6	2014 May
Other respiratory viruses			
Rhinovirus	35 (19.8)	10	2013 Oct
Parainfluenza viruses 1–4	5 (2.8)	1	NA
Human metapneumovirus	4 (2.6)	2	2014 May
Respiratory syncytial virus (A and B)	4 (2.6)	1	NA
Enterovirus	1 (0.6)	NA	NA
Adenovirus	1 (0.6)	NA	NA
Bocavirus	0	NA	NA
Human CoVs			
Human CoV OC43	6 (3.4)	3	2014 Feb
Human CoV 229E/NL63	2 (1.1)	1	NA
MERS-CoV	0	NA	NA
Bacteria			
* Chlamydophila pneumoniae*	1 (0.6)	NA	NA
*Legionella* spp.	1 (0.6)	NA	NA
* Mycoplasma pneumoniae*	0	NA	NA

*CoV, coronavirus; MERS, Middle East respiratory virus; NA, not applicable. †Among the 177 returned travelers, no respiratory pathogen was found for 88 (49.7%). Among the remaining 89 (50.3%) returned travelers, at least 1 respiratory pathogen was found; 12 (6.8%) of these persons had viral co-infections. Among the 12 co-infections were 8 rhinovirus co-infections (4 persons with influenza A and 1 each with influenza B, enterovirus, CoV OC43, parainfluenza); 1 influenza A–CoV OC43 co-infection; 1 influenza B–respiratory syncytial virus; 1 CoV 229E/NL63–adenovirus; and 1 CoV 229E/NL63–human metapneumovirus co-infection. ‡Comprises all reported infections, including viruses that were involved in co-infections.

No specimen submitted to the PHOL tested positive for MERS-CoV. Given the relatively low volume of travelers arriving to Canada and Ontario from MERS-CoV–affected areas (0.6% of total global travel from MERS-CoV–affected areas entered Canada during June–November, 2012, and <50,000 nonresident travelers entered Ontario from affected countries in 2012 [*11*,*12*]) and lower rates of human-to-human transmission, risk of importation to Ontario and subsequent local spread is likely low (*1*,*13*).

## Conclusions

Although the risk for MERS-CoV importation is low, respiratory virus infections acquired abroad or locally after returning to Canada might be relatively high and consistent, occurring in 87 (49.2%) of 177 PUIs during the study period. Most influenza B cases were detected shortly after the 2014 Ontario peak (PHOL, unpub. data). Furthermore, 75% of PUIs with influenza B reported symptom onset within 4 days after their return, possibly indicating local acquisition. Similarly, PUIs with enterovirus or rhinovirus detected probably acquired disease in Canada, given the short incubation period (mean 1.9 days) of rhinovirus (*14*).

Because limited information about clinical severity or outcomes was reported to PHOL, we were unable to report on the clinical spectrum of PUI presentation. Furthermore, pathogens were not identified for all samples, possibly because of delays between symptom onset and specimen collection, sampling technique, or other factors.

The number of PUIs with influenza (41 [23.2%]), whether acquired locally or abroad, is of particular concern. Unnecessary identification of PUIs could be avoided with more comprehensive vaccination coverage. Influenza vaccination should be a priority for all persons and should be recommended by health care practitioners who advise travelers. In addition, surveillance should continue for other respiratory pathogens so that their effects on health systems, when they co-circulate with emerging pathogens with similar clinical presentation, can be better understood.
